# Jaw muscle and joint psychophysics—relevance for clinical orofacial pain practice and research. A narrative review

**DOI:** 10.22514/jofph.2025.001

**Published:** 2025-03-12

**Authors:** Peter Svensson, Fernando G Exposto, Yuri Costa

**Affiliations:** ^1^Faculty of Dentistry, National University of Singapore, 119085 Singapore, Singapore; ^2^Section for Orofacial Pain and Jaw Function, Department of Dentistry and Oral Health, Aarhus University, 8000 Aarhus, Denmark; ^3^Department of Biosciences, Piracicaba Dental School, University of Campinas, 13414-903 Piracicaba, SP, Brazil

**Keywords:** Orofacial pain, Temporomandibular disorders, Psychophysics, Neuroscience, Pain assessment, Narrative review

## Abstract

Diagnosis of jaw muscle and temporomandibular joint (TMJ) pain has been greatly standardized with the development and implementation of the Diagnostic Criteria for Temporomandibular Disorders (DC/TMD). A significant part of the DC/TMD examination—pain on palpation and jaw movements—relies on psychophysical principles in the clinical procedures. Thus, it is essential that examiners are aware of the strengths and limitations of such techniques. Here we first review the background and psychophysical techniques used in the clinic and then discuss opportunities to apply both simple and more advanced modifications in research settings to further understand musculoskeletal pain mechanisms and signatures. The goal is to facilitate development of individualized treatment and precision medicine for which a good starting point seems to be careful pain phenotyping where psychophysical testing may play a substantial role.

## 1. Overview of clinical characteristics of painful jaw muscles and temporomandibular joints

Painful temporomandibular disorders (TMD) are typically described as the most common type of musculoskeletal pain in the orofacial region with prevalence in the range of 10–15% and more frequent in women than in men [1]. It is recognized that painful TMDs continue to be a challenge for both patients, health care professionals and society with significant impact on wellbeing, quality of life, mood, sick leave and societal aspects despite ongoing research efforts for decades [2, 3, 4].

As a way of standardizing the diagnosis of TMD both in the clinic and for research projects, the Diagnostic Criteria for TMD (DC/TMD) [5] was developed based on similar principles as the original Research Diagnostic Criteria for TMD (RDC/TMD) [6]. Pain is an important cause for patients seeking help and treatment and it is also important in establishing a diagnosis [7]. TMD pain is furthermore associated with impaired jaw function, quality of life and emotional challenges leading to a negative impact on psychosocial function [4, 8]. Although pain is a significant problem there are numerous ways in which pain presents for different patients. Pain may occur at rest (spontaneous) and may be provoked by jaw function or jaw movements (evoked). For a DC/TMD pain diagnosis both evoked and spontaneous pain must be present. However, this may lead to challenges in diagnosing patients that only have evoked muscle or temporomandibular joint (TMJ) pain such as during chewing or clenching.

Also, psychosocial distress can modify the presentation of TMD pain which furthermore may be influenced by cultural and ethnic factors [9]. Importantly, pain often varies in intensity leading to the necessity of evaluating both the least, the worst and the average pain levels over a period, typically in the last month [5]. Adding semantic, gender, cultural and societal dimensions to the understanding of pain is important as not all patients interpret pain in the same way [10, 11]. The DC/TMD instructions are designed to include only “pain” and disregard other unpleasant non-painful symptoms, but patients may use words such “tender” or “stiff” or “fatigued” to describe their complaint [12]. Whilst the strict focus in history on pain may help to filter the complaints, it may still be important to clarify with the patient if unpleasant but non-painful symptoms are associated with significant interference with function, mood and quality of life.

As part of a comprehensive clinical examination an assessment of the shape, size and consistency (*e.g.*, hardness, stiffness) of the jaw muscles is also needed and for the TMJ it is important to note if clicks, pops or grating sounds can be felt on standardized jaw movements in addition to swelling, redness and heat. We will not go further into the TMJ sounds or signs of inflammation but focus solely on the muscle and TMJ examination based on palpation. For decades it has been a controversial issue if so-called trigger-points could be identified in jaw muscles and if they are solely responsible for the phenomenon of referred pain, and if when managing them a twitch response is required to obtain a therapeutic response [13, 14, 15, 16, 17]. There is agreement that muscles can vary in texture and that palpable bands may be identified but there are also concerns about the reproducibility of such findings. Ultrasound imaging and Magnetic Resonance Imaging (MRI) is proposed to help identify the anatomical substrate of trigger points and taut bands although there is a clear discrepancy in the clinical location and ultrasound identified locations [18]. Indeed, a recent review suggested that there may only be weak evidence in favor of changes in instrumentally assessed muscle hardness in patients with painful TMDs [19].

What most clinicians and researchers will agree on is that the jaw muscles and TMJ need to be palpated by the examiner. Hence this is one of the diagnostic criteria for myalgia and arthralgia in the DC/TMD [5]. Also, palpation of musculoskeletal tissues in other conditions like osteoarthrosis, fibromyalgia, low back pain, *etc.* is the norm. It is therefore of crucial importance to have a good understanding of the principles of muscle and TMJ palpation.

## 2. Psychophysical principles

It may not be obvious at first glance that jaw muscle and joint palpation is, indeed, a psychophysical technique although quite simple and unsophisticated (Fig. 1).

**Fig. 1.** Overview of psychophysical principles for assessment of jaw muscle and temporomandibular joint pain. The stimulus (left black arrow) is an essential first step with options for “a” manual palpation, “b” palpometers, “c” pressure algometers, “d” dynamic roller algometers. The temporal features (t) and intensity (i) of the stimulus should be characterized, for example as “a” single but variable stimulus, “b” single and more stable stimulus, “c” repeated stimuli with known frequency or “d” as a single but prolonged stimulus. The response (right black arrow) is based on patient reports: Yes/No pain, numerical rating scales (NRS), visual analogue scales (VAS), verbal descriptor scales (VDS) or cross-modality matching (CMM). Applying different stimulus intensities allows construction of stimulus-response curves (S-R). Pressure pain thresholds represent another option for assessment of patient outcomes. Stimulation of heterotopic sites, *e.g.*, the hand may be considered a control for stimulation within the painful region and may allow for conditioning stimulation in order to assess conditioned pain modulation (CPM) responses. The psychophysical procedures may constitute an important part of phenotyping patients with musculoskeletal pain conditions including TMD pain in combination with measures of psychosocial distress (*e.g.*, Risk of Pain Spreading—ROPS and algorithms such as Rapid OPPERA Algorithm—ROPA). fMRI: functional magnetic resonance imaging; PET: positron emission tomography; NIRS: near infrared spectroscopy; EMG: electromyography.

Psychophysics can be described as the science behind how physical stimuli from the environment can be decoded and interpreted in terms of perceptual reports by the individual (for an overview see [20]). Psychophysics is abundantly used for various medical purposes, for example, testing hearing, vision, taste, smell and somatosensory function. An exogenous stimulus is provided, and the test subject will respond: “Yes I hear/see/taste/smell or feel the stimulus” [20]. In dentistry we have for decades tested the sensitivity of the periapical ligament by a percussion test (tapping mechanical stimuli) or the vitality of the tooth pulp by electrical or thermal stimulation. Without the response from the test subject the test cannot be interpretated in a clinical meaningful manner. Likewise, we have for a long time palpated the jaw muscle and TMJ by applying digital pressure, usually with the index finger and instructing the patient to respond to the question: “Was the pressure I applied to your muscle or joint painful—yes or no” [5, 6].

There are at least two important aspects of these simple psychophysical tests (see, *e.g.*, [21, 22, 23]). The first being related to the standardization of stimulus. It should be distinct, evoke a clear and natural sensation, be reproducible and not cause tissue harm. The control of stimulus intensity is of paramount importance because there is a well-established relationship between stimulus intensity, neuronal response and perceived magnitude of the stimulus [20, 21]. Also, the standardization of the duration of the stimulus is essential because differences in perceived magnitude of the stimulus may occur due to temporal summation (TS) and sensitization or adaptation and habituation. Both the RDC/TMD and DC/TMD specified the palpation pressure and time duration that should be applied to the various jaw muscles and TMJ. Perhaps surprisingly, palpation of cranial or cervical muscles is not essential for headache classification in the International Classification of Headache Disorders (ICHD-3) [24].

The other key aspect is related to instructions to the patient or the test subject that should be clear and unambiguous on how to respond to the stimulation [21]. In the simplest manner the response can be binary: “Yes—it was painful” or “No—it was not painful”. Slightly more sophisticated response assessment could be ordinal scales: “it was just barely painful, slightly painful, moderately painful or strongly painful”. From there it is a small step to apply scales with ratio-interval properties, *e.g.*, on a numerical rating scale (NRS) from 0 to 10 or a 10 cm visual analogue scale (VAS) or more sophisticated but seldom used in clinical research a verbal descriptor scale (VDR) [25]. Because more sophisticated response assessments have not been systematically investigated it is unclear to what extent they could improve the diagnostic process or guide better pain management. In addition, the response of the test subject can also be conceptualized as a “threshold”, for example, a sensory detection threshold can be defined as the lowest stimulus intensity that barely can be perceived in at least 50% of repeated stimulus presentations [20, 21, 22, 23]. Commonly, a pain threshold is defined as the lowest stimulus intensity that can be perceived as barely painful whereas the pain tolerance threshold is the highest stimulus intensity that the test subject is able or willing to accept. For further description of psychophysics and quantitative sensory testing (QST) in orofacial pain the reader can be referred to comprehensive reviews on the topic [21, 22, 23, 26].

A classical but common mistake may be to ask: “Did you feel anythingwhen I touched your muscle?”. In psychophysical terms this represents a detection threshold and not a pain threshold so as per the RDC/TMD and DC/TMD it is important that patients are informed only to report if the applied pressure stimulus was or was not perceived as painful. In the ICHD-3 pericranial tenderness is assessed through palpation and a system called the Total Tenderness Score (TTS) is applied [27]. However, the scale used for the TTS does not allow for ratings in both the painful and non-painful ranges, which could lead patients to label uncomfortable sensations as painful, even when they are not. Additionally, the inconsistent use of terms such as “tenderness”, “discomfort” and “pain” within this scale may result in varying outcomes, as has been observed with mechanical pain thresholds [28]. The applied force is also often not standardized, and when it is, they differ between studies or are not specified [29, 30, 31]. Lastly, there appears to be considerable variation in the muscles assessed across studies, potentially causing inconsistent results depending on which muscle groups are evaluated. This leads to results such as the masseter muscle which has a lower pressure pain threshold (PPT) than the trapezius muscle having a lower tenderness score than the trapezius muscle (**Supplementary Fig. 1**).

In a slightly broader perspective, the various jaw movements, *e.g.*, maximum opening, protrusion and lateral excursions, examined in the DC/TMD can be considered a natural (endogenous) stimulus of receptors other than nociceptors, such as proprioceptors and mechanoreceptors located in the musculoskeletal tissues including the skin and oral mucosa. In fact, one study employed repeated maximum jaw opening in patients with TMJ arthralgia and noted TS effects on the movement-evoked pain [32]. Therefore, it is essential to provide distinct instructions to the patient on “opening as wide as possible even if it is painful” and to standardize the number of times the jaw movement is repeated such as recommended in the DC/TMD [5].

In summary, this section has argued that the clinical examination per the DC/TMD involves a significant psychophysical test where stimulus (palpation pressure, jaw movements) needs to be standardized as much as possible and the response criteria carefully explained to the patients. In the following paragraphs, we will explore the current DC/TMD criteria in greater detail, focusing on pain, familiar pain and referred pain. This will lead to further discussion on trigger points, tender points, taut bands and twitch responses, all examined through manual palpation.

## 3. Manual palpation

There are several advantages in the detailed specifications for manual palpation per the DC/TMD [5]. The instructions to the patients are clearly outlined and it is explained that only painful responses but not tenderness or soreness or unpleasantness should be reported. The examiner is instructed to be calibrated by applying pressure on a scale to identify the correct level of pressure, *e.g.*, 0.5 kg or 1.0 kg. The clinical relevance of this calibration procedure has nevertheless not been formally tested (see also next paragraph). Another strength of the DC/TMD is that the specific regions of the jaw muscles and TMJ are clearly described, *e.g.*, the masseter muscle is divided into a superior, middle and inferior part each with 3 sites for manual palpation based on identification of the outline of the muscle during repeated contractions. The temporalis muscle is divided into the anterior, middle and posterior part with 3 sites for manual palpation and again after identification of the outline of the muscle during repeated contractions. The TMJ pole is targeted at 0.5 kg for 2 seconds and dynamic palpation around the pole at 1.0 kg over 5 seconds. Furthermore, muscle palpation should last 2 seconds to assess pain and familiar pain, and 5 seconds to assess for the presence of referred pain. It could be argued that the specific palpation pressures and durations are arbitrarily decided, and we will discuss this in more detail in the paragraph on pressure algometers. It could also be argued that due to the concept of “familiar pain”, introduced in the DC/TMD, as long as the palpation stimulus triggers pain, the diagnostic accuracy remains unchanged, regardless of whether the examiner is calibrated or not. Nevertheless, the inclusion of the “familiar pain” concept in the DC/TMD may account for the increased sensitivity and specificity values of the different myalgia diagnoses as well as arthralgia when compared to the RDC/TMD [5]. Once patients have understood the concept of familiar pain on palpation and jaw movements, the outcome of the examination is better linked to the chief complaint of the patient. Other diagnostic systems could consider including the concept of familiar pain on palpation.

Finally, the concept of referred pain within the DC/TMD is explained as pain that did not stay under the palpating finger but spread outside the boundaries of the palpated structure on the same side [5]. There is an attempt to distinguish between spread and referral of pain but spreading pain has not been specified in terms of sensitivity and specificity in the DC/TMD and the difference in mechanism between the two phenomena as well as the importance to patient management in their distinction has not been clarified. One possibility could be that spreading pain may be the clinical manifestation of an increase in receptive fields of second-order wide-dynamic-range neurons in the trigeminal sensory nuclei complex and referred pain because of an activation of convergent afferent input onto such second-order neurons [33, 34, 35]. In addition, release of neuroinflammatory substances such as substance P, Calcitonin-Gene-Related Peptide may play a role in these phenomena [35]. Referred pain is both a reliable and valid finding on standardized palpation and included in the DC/TMD to link to the more general concepts of myofascial pain and the debatable concept of trigger points. Note that the DC/TMD does not include a description of active or latent trigger points as per the classical descriptions from Simons and Travell [36] and there are no criteria for taut bands or twitch responses.

Both clinical experience and a substantial number of scientific reports have beyond any reasonable doubt established that (jaw) muscles can refer pain to typical sites and regions in an almost pathognomonic pattern [36, 37, 38]. As such, it is important to assess for referred pain from the masticatory and cervical systems in patients with orofacial pain to rule out that for example pain felt in the teeth is not being referred from the above-mentioned structures [39]. Referred pain has also been linked to more widespread pain [40] and may be an indicator of more profound nociceptive activity. No studies have so far demonstrated differential treatment effects on myofascial pain with referral when opposed to myalgia without referral. In addition, the reliability of manual palpation for the identification of trigger-points and taut bands has been deemed to be unreliable for both nonexperts and experts [41, 42] and studies using ultrasonography to identify hypo-echoic structures do not show a convincing overlap [18]. Original studies with micro-dialysis were able to identify low pH, release of neurotransmitters on palpation and local twitch responses [43, 44] but such studies have not been replicated, had an extremely small sample size and the used methodology has recently come under scrutiny [45]. It seems safe to conclude that referred pain occurs on standardized stimulation of both jaw muscles and TMJ, but the exact pathophysiological implications and possible anatomical substrate of trigger points and taut bands remain uncertain. It should also be noted that referrals are not always described as painful as they may also be described as a nonpainful sensation which has been clearly demonstrated with appropriate rating scales [46]. It is not yet known if there are any clinical implications for differences between referred pain and referred sensations.

Muscle and TMJ palpation of children and adolescents have been discussed in Delphi reports to be in need of modification regarding the applied pressure and number of test regions, as well excluding the assessment of referred pain [47, 48]. However, the referred pain phenomenon seems also to be reliably identified in children and adolescents with similar patterns of referral as adults [49].

The RDC/TMD suggested grading the pain response on palpation as no pain/mild/moderate/strong pain (ordinal scale) but this was simplified in the DC/TMD to the binary response—yes/no painful. For clinical purposes this simplification does not seem to lead to loss of information that may be important for the diagnosis and/or management of patients. However, grading of palpation responses may reflect clinical severity and has been advocated in clinical practice [50].

In summary, the DC/TMD and the new brief DC/TMD [51] offer unique opportunities to perform standardized examination of jaw muscles and TMJ thanks to the operationalized criteria and detailed instructions. Similar high-quality guidelines will also emerge for children and adolescents [48, 52]. However, despite the intention to standardize the psychophysical examination, studies have shown considerable variability in the pressure stimulus. This variability will be discussed in the following paragraph.

## 4. Palpometers

In order to standardize the palpation pressure, at least two innovative attempts have been made. The first was based on a pressure foil/sensor attached to the index finger and connected to an electronic display that could guide the examiner to reach the intended amount of pressure [53, 54]. The electronic palpometer demonstrated good psychophysical properties and could reliably establish stimulus-response curves and when applied to patients with tension-type headaches demonstrated substantially higher tenderness scores and qualitative differences in the evoked experience when compared to non-headache patients [55]. The disadvantage of this prototype was the need to have a sensor attached to the finger and the need to calibrate arbitrary units into kg/kPa. This attempt seems not to have led to lasting changes in headache clinics other than suggestions to be included in future laboratory assessments of subtypes of headaches [56].

Another more recent attempt is based on a simple mechanical device consisting of a spring coil with a predetermined force and a small stamp touching the finger of the examiner and thereby providing a mechanical feedback signal that the calibrated force had been reached [57, 58, 59] (Fig. 1). Separate devices with unique and calibrated spring coils corresponding to 0.5, 1.0, 2.0 and 4.0 kg and a silicon covered 1.1 cm diameter probe would then cover an appropriate range of forces to be used in clinical examination of patients with various types of musculoskeletal pain disorders including painful TMDs. Methodological studies clearly demonstrated that the use of mechanical palpometers significantly reduced the variability of repeated force applications (increased precision) and increased accuracy, *i.e.*, closer to the target force than manual palpation with either the index or third finger, left or right hand and on soft or hard surface [57, 59, 60]. Furthermore, studies indicated that novice examiners with little or no prior experience with manual palpation performed as well as experts when a palpometer was used [61]. A recent study was not able to identify significant differences in actual applied force between male and female examiners with the use of a 1.0 kg palpometer [62] and found no significant effect of prior calibration. So far, no studies have focused on the systematic training to target the recommended palpation pressures and if experienced clinicians would perform better than inexperienced clinicians. As the DC/TMD provides description of a dynamic, *i.e.*, 5-second moving palpation around the TMJ, methodological studies have also demonstrated increased accuracy and precision with a 1.0 kg palpometer compared with manual palpation but also that palpation duration is often shorter than the targeted duration of 2 and 5 seconds of palpation [60]. This seems to be inherent in the ability of the examiner to maintain a constant force overtime despite the mechanical feedback.

Another way of using the palpometer has to do with assessing TS of pain, a possible measure of central sensitization, in deep tissues such as muscle. The German Network Protocol for Neuropathic Pain includes a procedure for calculation of TS of painful pinprick stimuli applied to the skin termed the Wind-Up Ratio (WUR) [63]. This can be modified with the use of the palpometer, *e.g.*, a single stimulus versus 10 repeated stimuli at different frequencies and durations. The reliability of this procedure in terms of the generated mechanical forces is far better than if manual repeated stimuli a being attempted suggesting that a novel measure of deep sensitization can be accomplished by adapting the palpometer to an already existing protocol. The above-mentioned studies provide good evidence that it is possible to standardize the pressure stimulus thereby reducing the overall variability of the psychophysical test. Inherent in such tests is the variability associated with the patient/test subject’s perception and response. Maintaining sufficient interstimulus periods, repeating the same stimulus multiple times [3, 4, 5] and using a central tendency measure (*e.g.*, geometric mean or average) to calculate thresholds or ratings in addition to checking for attention and alertness and cooperation of the test subject seems essential to further minimize psychophysical variability [21, 63].

From a rating point of view, the palpometers can be applied in accordance with the DC/TMD description (binary response) but can also be further rated using for example 0–10 NRS which possess ratio-interval properties. However, a modification was suggested in order to include non-painful pressure sensations so a 0–50–100 NRS can accommodate both non-painful pressure sensations and painful pressure sensations based on 0 = no sensation at all, 50 = just barely painful and 100 most painful imaginable [64]. Stimulus-response curves have demonstrated good psychophysical properties with near-linear relationships in the range of 0.5–4 kg of stimulation [58, 59, 65]. Obviously, such NRS data can also be dichotomized into non-painful and painful responses and may allow for estimation of a pain threshold based on stimulus-response curves.

From a clinical perspective the palpometers can further be modified to meet different criteria for applicability, *e.g.*, around the TMJ with the dynamic palpation technique where the flat stimulation probe can be replaced by a ball allowing a smooth movement [66]. Moreover, add-on extensions in order to reach, for example, the temporalis tendon can be used [67, 68] and probes with smaller diameters and extensions can be applied for intraoral use of the gingiva obviously changing the pressure as the stimulus area is reduced [69]. Such so-called semi-quantitative techniques can be applied, *e.g.*, for assessment of intraoral neuropathic pain conditions in the clinic [22].

From a mechanistic point of view, the increased standardization of the pressure stimulus with the use of the palpometers has allowed systematic studies and description of the phenomenon of referred pain. However, an important finding is that referred pain can be triggered in healthy non-painful participants from the masseter, temporalis, temporal tendon and TMJ with a monotonic increase in prevalence with increasing palpation force and duration [46, 66, 68, 70, 71]. This observation is also in line with a series of studies using intramuscular or intraarticular painful stimulation of jaw and neck structures [72, 73, 74, 75]. The observation of consistent referral patterns in otherwise non-painful individuals suggests that referred sensations are naturally linked to painful stimulation of deep tissues like muscles and joints, *i.e.*, referred pain and sensations have been suggested to be an epiphenomenon and not a distinct marker of pathophysiology [76]. In fact, experimental sensitization of jaw muscles with intramuscular injections of Nerve Growth Factor did not yield a higher prevalence of referred sensations than a control isotonic saline stimulation [77, 78]. It has also been demonstrated that referred sensations can be shifted towards a preceding noxious input from the retromolar region, *i.e.*, referred pain may be dynamic and state dependent [79]. Endogenous pain modulation protocols can also be applied to modify referred pain and sensations suggesting alterations at the central nervous system level rather than at the periphery may be responsible for this phenomenon [80]. These are just recent examples showing that if the stimulation technique is sufficiently standardized and reproducible then insights into the physiology and pathophysiology of deep tissues can be obtained even with relatively simple psychophysical techniques.

The main advantage of the palpometers may be the simplicity of use and that they do not require expensive equipment which otherwise can be used in QST protocols, *e.g.*, pressure algometers. This is the next level of psychophysical testing in TMD pain patients and will be described in the following paragraph.

## 5. Static pressure algometers

Various types of pressure algometers have been developed to evaluate musculoskeletal pain sensitivity (Fig. 1). Such algometers should meet the criteria for standardization of the pressure stimulus as well as the response which typically is determined as a PPT. The PPT is defined as the amount of pressure that the test subject barely perceives as painful. It could be suggested that in line with the DC/TMD the explanation of PPT is further qualified by the use of the term familiar pain. For most pressure algometers, the examiner applies the force, for example, with a constant increase in pressure per time unit (30–50 kPa/second) with use of visual feedback and the test subject pushes a response button once the PPT has been reached. This procedure is often repeated 3–5 times and the average PPT is calculated and used for further analyses [63]. Some studies have also tested the pressure pain tolerance (PPTol) as the maximal pressure that the test subject is willing or able to tolerate. In this procedure there is a risk for sensitization of the underlying tissue due to high pressure stimulation and potential tissue damage. Methodological studies have demonstrated both the reliability and importance of standardization of stimulus parameters, *e.g.*, probe size and edges, pressure application rate, intervals between repeated measures, jaw gapes, level of muscle contraction, *etc.* [81, 82, 83, 84, 85, 86]. Interestingly, studies have also shown that anesthesia of the skin has some [87] or no impact [88, 89] on the PPT suggesting that PPT represents a measure of deep musculoskeletal pain sensitivity with minor contribution from the skin if probe diameters are 10 mm or more. The impact of the amount of subcutaneous fat and parotid tissue overlying the posterior parts of themasseter muscle seems to not have been systematically investigated so far. In terms of gender differences, PPTs have been used to consistently demonstrate that women have lower PPTs than men [90] and it has been speculated to what extent this observation reflects biophysical properties (*e.g.*, smaller muscles, thinner skin) or differences in nociceptor density or individual differences in the interpretation of pressure pain. Importantly, the gender of the examiner seems to be another significant factor for standardization [91].

Most pressure algometers can easily be applied to different body regions including the cranial region with the exemption that not many studies have tried to measure intra-oral PPTs [92, 93]. More sophisticated pressure algometers with computer control of pneumatic devices to further standardize the pressure stimulus delivery for extremities have been developed (*e.g.*, [94]) but may not be applicable to the cranial region.

A psychophysical variation of the PPT measurement, the so-called stimulus-response curves can be constructed with the use of pressure algometers on the jaw muscles [85]. The advantage may be that the slope of the stimulus-response curve contains information about the gain in the somatosensory processing and relies on more than just one single measure of a pain threshold.

It is a consistent finding that PPTs of the jaw and cranial muscles are reduced in patients with myofascial TMD or TMJ arthralgia compared to matched controls [73, 82, 85, 95, 96, 97]. Reduced PPTs of pericranial muscles in various headache conditions are also a consistent finding [98]. Interestingly, many studies have shown reduced PPT at body sites remote from the painful region which has been interpretated as an indication of more widespread hypersensitivity (*e.g.*, [73, 95, 96, 97]). There remains a discussion on whether such findings are explained by central sensitization which is a specific neuronal process observed in wide dynamic range neurons in the spinal cord (or brainstem) but which may not necessarily account for reduced PPTs at non-painful body sites [99].

An intriguing point mentioned above for the DC/TMD criteria is the specific amount of pressure recommended for manual palpation. Indeed, studies with the use of PPTs have tested cut-off threshold for “normal” and “painful” sensitivity. Conti and colleagues [100, 101] in two studies addressed this issue for patients with TMJ arthralgia and myalgia and based on Receiver Operating Characteristics (ROC) and analyses of the sensitivity and specificity they recommended that for TMJ pain the cut-off should be set at 1.36 kg and for myofascial TMD pain at 1.5 kg for the masseter muscle and between 2.47–2.77 kg for the temporalis muscle depending on the site [100, 101]. There may be practical difficulties reaching such targets with sufficient accuracy and an inherent problem based on diagnostic criteria and case definitions.

In summary, PPTs are commonly used in research projects to demonstrate increased sensitivity of the jaw muscles and TMJ in various types of TMD patients. Primarily costs and physical size of the pressure algometers continue to be the main disadvantages in more widespread implementation in clinical practice. PPT assessed as described above can be considered to represent static mechanical allodynia and more recent attempts have been devoted to also test dynamic pressure pain sensitivity.

## 6. Dynamic pressure algometers

Relatively simple devices based on a similar principle as the mechanical palpometers, *i.e.*, a calibrated spring coil but with a 1 cm wide wheel as the probe allowing to steadily move the device with a fixed stimulus intensity (provided by the examiner) over the surface of a muscle [102] (Fig. 1). This has been tested on the temporalis muscle in different headache conditions compared to matched healthy controls (*e.g.*, [103]). The dynamic pressure sensitivity can be assessed as the dynamic pressure pain threshold by applying different pre-set rollers, *e.g.*, from 350 g to 5000 g with a wheel diameter of 35 mm and a feedback-controlled speed of 1 cm/second. The advantage of this innovative technique is that a larger part of the muscle can be examined and described. It may even be possible to synchronize pain ratings, *e.g.*, on electronic VAS and to link changes in pain sensitivity to specific sites of the muscle. Approaches like this may be useful to re-examine the concepts of trigger points or at least for a better spatial mapping of target muscles like the temporalis and masseter muscle. However, depending on the underlying tissue this technique may not easily be implemented for, *e.g.*, the sternocleidomastoid or smaller jaw and neck muscles.

## 7. Spatial mapping

The dynamic pressure algometers represent a psychophysical attempt to incorporate the spatial or topographic dimension of musculoskeletal pain into a better understanding of characteristics and potential underlying mechanisms in pain patients. Another attempt is based on standardized stimulation of multiple sites guided by, *e.g.*, 1 × 1 cm grids to systematically cover the entire muscle or TMJ (Fig. 1) (see also [81]). As mentioned, the DC/TMD recommends covering the masseter and temporalis muscles with 3 × 3 = 9 sites. Methodological studies have applied 3 × 5 grids over the masseter and temporalis muscles and repeatedly stimulated each grid with a fixed stimulus intensity, *e.g.*, 0.5/1.0 or 2.0 kg or measured the PPT at each site [104, 105, 106, 107]. Obviously, this procedure provides a more fine-grained analyses of the entire muscle sensitivity and not simply relying on binary responses for the entire muscle. The advantage lies in the opportunity to determine more composite measures of muscle sensitivity, for example, the peak location, *i.e.*, most sensitive parts can be determined using center-of-gravity calculations or the heterogeneity, expressed as entropy. It might also be feasible to identify the number of sites associated with a familiar pain sensation as another measure of pain sensitivity. Studies have demonstrated that a distinct painful input provided by injections of glutamate or Nerve Growth Factor (NGF) into the masseter muscle significantly increases the entropy as an indication of overall sensitization of the muscle [65, 105]. This technique is now being implemented in studies of TMD pain patients (Fig. 2).

**Fig. 2.** Preliminary data showing color-coded heat plots of perceived pain on a 0–50–100 numerical rating scale (NRS) following stimulation with 1.0 kg palpometer at each of the grids in the temporalis and masseter muscle in patients with myofascial TMD pain (n = 28) and matched control individuals (n = 28). Average of the two sides. The estimated entropy was 1.1 + 0.3 and 1.2 + 0.3 in the temporalis and masseter muscle in TMD patients and 0.9 + 0.4 and 1.0 + 0.3 in the controls indicating larger inter-grid variation in TMD patients. Courtesy of dr. Juan Fernando Oyarzo Sardiña.

From a larger perspective it seems important to observe the spatial distribution and spread of pain over the body termed Risk of Pain Spreading (ROPS) [108]. From a regional point of view, it may similarly be important to account for the spatial spread of TMJ pain or spread of masseter and temporalis pain. Psychophysical modifications and standardization of pressure stimuli may be useful in this respect and represent an era with more focus on “topoalgia”.

## 8. Temporal summation

Repetitive stimulation with distinct stimuli can lead to an increase in the perceptual response evoked as briefly mentioned above (Fig. 1). A stimulus frequency of 1 Hz seems to reliably trigger TS with mechanical stimuli [109]. Alternatively, for muscle assessment it is possible to observe increased sensitivity when the pressure stimulus is held for a longer time, *e.g.*, 10 s repeated 10 times (Fig. 3).

**Fig. 3.** Preliminary data showing temporal summation using a 1.0 kg palpometer applied to the anterior temporalis (average of the right and left side) and calculated as the difference between the tenth minus first stimulus (delta values). Pressure stimuli were applied for 10 seconds repeated 10 times. Boxes indicate the mean (middle line) and the standard error of the mean. Whiskers indicate the 95% confidence interval of the mean. There was a significant difference between patients with painful temporomandibular disorders (TMD, n = 30) and healthy participants (n = 30) (*p* = 0.025). NRS: numerical rating scale (0–50–100).

TS is the perceptual correlate of wind-up of neuronal responses and has primarily been studied on TMD pain patients with the use of thermal stimuli applied to the forearm or hand [110, 111]. Also, mechanical stimuli applied to the finger can cause perceptual increase of repeated mechanical stimuli with increased responsiveness in TMD patients when compared to matched controls [112]. Another characteristic of TS is that pain may outlast the physical stimulation, and the aftersensation can be used as another measure of increased sensitivity in pain patients. It should be noted that aftersensations can also be observed after single painful stimuli [46]. Preliminary studies have applied repetitive palpometer stimuli in TMD patients and demonstrated increased responsiveness of the jaw muscles and TMJ. It is a reliable phenomenon and possibly represents a measure of central nervous system sensitivity although not a specific measure of neuronal hyperexcitability (for a discussion see: [99, 113, 114]). With appropriate modifications of the psychophysical procedures also modulatory pain mechanisms can be studied which will be discussed in the following paragraph.

## 9. Conditioned pain modulation

The most common studied type of pain modulation is the so-called conditioned pain modulation (CPM) effect (Fig. 1) whose animal counterpart diffuse noxious inhibitory control (DNIC) first was described in classical animal studies [115]. Basically, the principle is that a painful stimulus presented as a conditioning stimulus will inhibit the presentation of another painful test stimulus elsewhere on the body [116]. Recommendations on how to standardize CPM protocols have been proposed and also applied in the orofacial region and on TMD pain patients [97, 117]. Originally, it was believed that CPM effects could be impaired in TMD pain patients, but systematic studies have found relatively weak evidence in favor of this suggestion [99]. CPM effects appear to be sensitive to methodological issues but also expectations, placebo and nocebo responses [118]. Therefore, as for any other psychophysical tests, standardization of specific instructions for test subjects is mandatory. Pressure stimulation is often used as the test stimulus and both painfully hot or cold water immersion of arms or legs can be used as the conditioning stimulus. In addition, pressure tourniquets around the arm and a special designed head-screw device have been used as conditioning stimuli for reliable induction of CPM effects [119]. The value of CPM assessment has been suggested to be the possibility of predicting treatment efficacy [120]. However, results have been contradictory with some studies showing a less efficient CPM is associated with better treatment outcome [121] while other studies have shown that a more efficient CPM is associated with better treatment outcome [122, 123]. CPM has been suggested to be a keystone pain mechanism [124] and further standardization should be prepared for application in the trigeminal pain system for example using palpometers or pressure algometers for standardized test stimulation.

Another type of endogenous pain modulatory system that has been characterized with the use of psychophysics is the so-called off-set analgesia [125]. This phenomenon is described as a disproportionate large reduction in perceived intensity of a stimulus provided a small decrement in stimulus intensity. It has primarily been shown with the use of thermal stimuli and apparently no studies have attempted to use mechanical stimuli. Off-set analgesia has also been described in patients with painful TMDs [126].

Studies on CPM and off-set analgesia will continue to develop and be valuable for a better understanding of some of the potential keystone pain mechanisms in painful TMD and contribute to the mosaic of factors involved in the modulation and processing of nociceptive function and central regulation including brain imaging.

## 10. Palpation-evoked brain signatures

Brain imaging has evolved tremendously over the last two-three decades and contributed to insights into the central processing in patients with painful TMDs [127, 128]. Several methodological advancements have been made in terms of the sophistication of image analyses, connectivity and network assessment and both spatial and temporal resolution of the brain responses. Nevertheless, brain imaging studies of pain rely heavily on psychophysical principles, *i.e.*, there must be a standardized stimulus for time-locked activation of the brain and the interpretation of neural signatures is meaningless without the test subjects rating or scores of the stimulus (Fig. 1).

A few brain imaging studies have excelled because of their use of advanced psychophysical principles. The first is a study in fibromyalgia (FM) patients exposed to pressure stimuli applied to the finger [129]. FM patients have consistently been shown to have lower PPT but instead of using stimulus intensities linked to the PPT an adjustment of the stimulus intensity to the perceived level was done, *i.e.*, FM patients were during the brain imaging stimulated with lower stimulus intensity but matched to the same perceptual level as controls. This allows for an interpretation of the neural signatures not tied to the stimulus intensity but to the perceptual level. A similar psychophysical approach was used in a recent brain imaging study on FM and osteoarthritis (OA) patients allowing to discriminate between a more sensory-discriminative neural signature for the OA patients and a more emotional neural signature with frontoparietal activity for the FM patients [130]. Similar attempts with implementation of sound, vision and taste psychophysical principles should be implemented for brain imaging studies in TMD patients in order to further understand for example the concept of nociplastic pain versus nociceptive pain.

In the final paragraph we will focus on psychophysical measures used as a risk predictor for onset and transition of acute to chronic pain.

## 11. Pressure pain as risk predictor and proxy of pain mechanisms

The Orofacial Pain: Prospective and Evaluation Risk Assessment (OPPERA) study included a variety of QST measures including PPT and demonstrated significantly higher pain sensitivity for first-onset TMD pain cases compared to controls [131]. Further analyses were, however, not able to robustly associate PPTs at baseline (*i.e.*, before onset of TMD pain) to subsequent development of TMD pain and suggested that PPTs had low predictive value and did not signify pathophysiological mechanisms linked to first onset TMD pain [132]. As such this might be in line with a recent study on Risk of Pain Spreading (ROPS) where psychosocial indicators (sleep, mood, stress) were strong and significant predictors whereas biological markers including measures of pain sensitivity appeared to play less of a role although marginally significant [108]. It seems that it would be overly naive to think that a single psychophysical measure like a PPT could encapsulate all the complexity of development and chronification of pain, but it may still be relevant to explore clinical applicable measures of musculoskeletal pain sensitivity as part of a comprehensive evaluation and risk assessment of patients which is the underlying suggestion in identification of keystone mechanisms. As for any other technique, it is important to understand the limitations and appreciate the host of factors that can influence psychophysical measures of pain and their interpretation [114]. This view is supported by the more recent findings from the OPPERA study using cluster analyses which demonstrated that PPTs in addition to measures of depression, anxiety and somatization provided 3 distinct clusters of TMD pain patients [133]. These clusters included a Pain Sensitive group, an Adaptive group and a Global Symptoms group. These findings were later replicated in a population of chronic overlapping pain conditions and patients from a tertiary pain clinic indicating that PPTs together with measures of psychosocial distress indeed have predictive potential [134] for TMD pain and potentially also for headache conditions [135]. This further prompts the suggestion to include PPTs or other measures of deep pain sensitivity as important and clinically feasible biomarkers and prognosticators but in concert with evaluation of psychosocial distress, *i.e.*, a pragmatic approach to identification of some keystones of pain mechanisms (Fig. 1).

## 12. Future developments and recommendations

The present narrative review has attempted to outline the role of psychophysical principles in both the clinical examination of TMD pain and in the research setting. Key issues are standardization of the stimulus (palpation pressure and duration) and the evoked response (rating or threshold) from the test subject. With relatively minor modifications it is possible to get further insight into the function of the central nervous system, *e.g.*, temporal and spatial dimensions, endogenous pain modulation, brain signatures and even with some restrictions also the prediction and assessment of risks. Fig. 1 provides an overview of possibilities for application of various psychophysical tests to further understanding of myalgia and arthralgia related to TMDs. Muscle and joint palpation remain a critical part of the clinical diagnostic process for musculoskeletal pain conditions and require attention and careful execution as highlighted in the instructions to the DC/TMD [5].

The DC/TMD has a well-founded focus on the major jaw muscles like the masseter and temporalis muscle and TMJ. In individual cases other jaw and cranial muscles may also be of importance to test and further standardization of, *e.g.*, the neck and cervical muscle examination might be clinical valuable considering the high degree of co-morbidity between orofacial and cervical pains and because of characteristic patterns of referral from the neck to the orofacial region. Also, intraoral palpation will need to be further standardized in order to reduce the variability of the applied stimulus. For example, palpation of the temporalis tendon might be of diagnostic importance in the differentiation of TMD pain and headaches.

Further incorporation of local anesthetic blocks in combination with palpation might also be useful to better characterize source and referred pain patterns. This calls for standardization of the procedure, *e.g.*, placebo control and again the specific psychophysical procedures applied.

In conclusion, significant improvement of diagnostic criteria has led to DC/TMD and further refinement summarized in the present review may pave the road for identification of keystone pain mechanisms and guidelines for more patient-centered treatment approaches.

## Supplementary Material

Supplementary material associated with this article can be found, in the online version, at https://files.jofph.com/ files/article/1887742892528549888/attachment/ Supplementary%20material.docx.


