# Supplementary material for: Jaw muscle and joint psychophysics—relevance for clinical orofacial pain practice and research. A narrative review
Source: J Oral Facial Pain Headache. 2025 Mar 12;39(1):1–14. doi: 10.22514/jofph.2025.001 (PMC11934744; doi:10.22514/jofph.2025.001)
Supplement: Supplementary file 1 [file Supplementary-material.docx]

Supplementary material


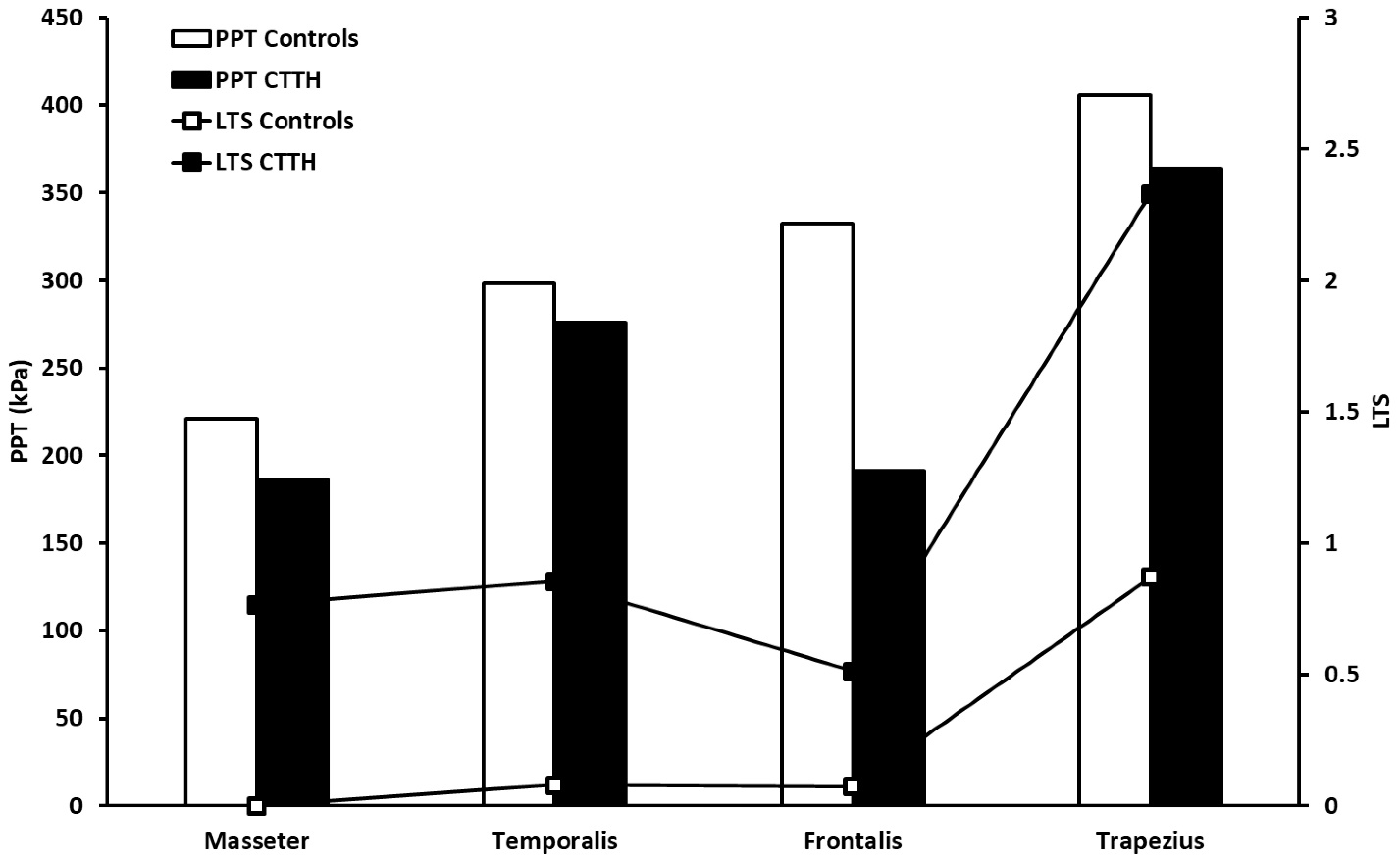


**Supplementary Fig. 1. Pressure pain thresholds (PPT) and Local Tenderness Scores (LTS) in chronic tension type headache (CTTH) patients and healthy controls.** The LTS data were retrieved from Bendtsen *et al.* [55] whereas the PPT data were taken from Andersen *et al.* [135] and Castien *et al.* [98]. The paradox is that the masseter muscle has the lowest PPT values (most sensitive) but the lowest LTS (least sensitive) in comparison to the trapezius muscle.
